# Impact of valve fenestrations and structural changes in homografts on the long-term outcome in the recipient

**DOI:** 10.1007/s10561-020-09886-5

**Published:** 2021-02-12

**Authors:** Ida Axelsson, Torsten Malm, Johan Nilsson

**Affiliations:** 1 Tissue Bank Lund, Lund University and Skane University Hospital, Baravägen 37, 22185 Lund, Sweden; 2 Department of Clinical Science Lund, Cardiothoracic Surgery, Lund University and Skane University Hospital, Lund, Sweden; 3 Pediatric Cardiac Surgery Unit, Children’s Hospital, Lund University and Skane University Hospital, Lund, Sweden

**Keywords:** Homograft, Right ventricular outflow tract, Tissue bank, Valve fenestrations, Long-term outcome

## Abstract

Homografts have long been used for right ventricular outflow tract (RVOT) reconstruction. Tissue banks struggle to meet the clinical demand of tissue, with insufficient donor availability and strict recommendations on tissue quality with high proportions of discards. This study analyzes the long-term outcome of patients receiving a homograft with small fenestrations of the cusps or other structural changes, to evaluate if minor impairment of the homograft affects the durability. Homograft characteristics and patient outcome were described. Follow-up was maximum 24 years. Structural changes of the homografts were analyzed in relation to patient outcome, using univariable and multivariable Cox proportional hazard regression. Between 1995 and 2018, 468 patients received 535 homografts in the RVOT in Lund. Median recipient age was 13 years. There were 137 (26.9%) reinterventions. Freedom from reintervention was 75.8% (95% CI 71.3–79.7%) at 10 years and 57.4% (95% CI 50.0–64.0%) at 20 years. Small fenestrations of the cusps, fibrosis of the cusps and minor atheromatosis of the vessel did not show any statistically significant impact on long-term outcome, hazard ratio = 0.46 (95% CI 0.11–1.87, *p* = 0.276) and hazard ratio = 0.80 (95% CI 0.25–2.56, *p* = 0.704). Minor structural changes of the homografts seem to be acceptable without affecting the long-term durability.

## Introduction

Homograft is the conduit of choice for reconstruction of the right ventricular outflow tract (RVOT) in children and adults with heart valve disease. The homograft show excellent haemodynamic performance, with low incidence of endocarditis (Van Dijck et al. 2015; Gröning et al. 2019). The durability of the homograft varies between individuals, but long-term follow-up of larger, heterogenous groups have shown 70–83% freedom from reintervention after 10 years (Bielefeld et al. 2001; Meyns et al. 2005; Skoglund et al. 2017; Romeo et al. 2019; Dekens et al. 2019). These numbers vary considerably depending on patient diagnosis, age and procedure. For example, patients who underwent the Ross procedure had 92% freedom from reintervention after 16 years (Da Costa et al. 2017), while a young patient group (< 1 year) with more complex diagnoses showed 38% freedom from reintervention after 10 years (Vitanova et al. 2014).

When collecting and preparing a homograft, several aspects have to be considered. The European Directorate for the Quality of Medicines and Health Care (EDQM) has developed recommendations for tissue banks, concerning matters such as assessment of donors and collection, preparation and decontamination of tissues (Keitel 2019). However, many recommendations rely on experience rather than scientific studies. Most studies in this area evaluate the performance of the homograft in relation to patient and homograft characteristics, where young patient age, young donor age, small homograft diameter, aortic homografts and heterotopic implantation of the homograft have been identified as risk factors for reintervention in repeated studies (Gerestein et al. 2001; Meyns et al. 2005; Brown et al. 2005; Boethig et al. 2007; Kalfa et al. 2011; Romeo et al. 2019; Dekens et al. 2019). Few studies look into the processing of tissue, and how that could affect the quality of the homograft.

One recommendation written by EDQM is describing which structural changes can be accepted in homografts used for transplantation. They describe that minimal presence of calcification, atheromatosis or fibrosis can be accepted, and that small fenestrations of the cusps can be accepted if they are located within the margins of the cusps lunulae or marginal of the cusps, as long as they do not induce regurgitation. They also state that large fenestration should not be accepted, but “large” is not further defined (Keitel 2019).

At the Tissue Bank in Lund, guidelines have been developed over time together with the national group of cardiovascular homografts in Sweden and in accordance with the guidelines from EDQM. These local guidelines state that soft atheromatosis of the vessel wall is accepted, as long as it is not widely spread. Fibrosis of the cusps are accepted if it does not affect the cusp motility. Small (less than 3 × 2 mm), peripheral fenestrations (close to the commissures) are accepted if they do not induce regurgitation.

The present study evaluates the long-term outcome of homografts with minor structural changes, with focus on small fenestrations, compared to homografts with no morphological deterioration. Experience say that there is no difference in performance between the two, but no scientific evaluation has been made. The primary objective was to evaluate if current guidelines on structural changes are accurate or not. A secondary objective was to describe the prevalence of homograft discard and reasons for discard at the Tissue Bank in Lund.

## Patients and methods

The Regional Ethical Review Board in Lund, Sweden, approved the study. Identification number of the application was 2017/133. All data was collected retrospectively.

### Donors and homografts

Homografts collected at the Tissue Bank in Lund between 1995 and 2018 were included in the study. The Tissue Bank in Lund distributes homografts to cardiac surgery centers in all Scandinavian countries except Finland. General criteria for heart valve donation was age below 65 years and weight above 5 kg, although a few older donors as well as some infants < 5 kg were accepted after closely reviewing medical history and other contraindications. Maximal ischemic time for non-heart beating donors (NHBD) was 48 h, with a recommended maximum of 6 h of warm ischemic time. The definition of ischemic time is time from circulatory arrest to heart explantation. NHBD was divided into two groups, with up to 24 h and > 24 h of ischemic time. In multi-organ donors and domino donors (recipients of a heart transplant), the heart was circulated until time of retrieval.

The preparation, antibiotic decontamination and cryopreservation of the homograft have been described elsewhere (Axelsson and Malm 2018). During preparation, the cusps and vessels are closely reviewed for damage and insufficiency. The homograft is turned inside-out to inspect the cusps. Fenestrations are measured with a sterile ruler. Small (< 3 × 2 mm), peripheral fenestrations are accepted, but homografts with larger fenestrations or fenestrations located centrally on the cusps are always discarded. The vessel wall is inspected for atheromatosis. Soft atheromatosis in smaller parts of the vessel wall is accepted. Homografts with ulcerated plaque, hard atheromatosis, atheromatosis that is loose from the vessel wall or widespread soft atheromatosis throughout the vessel wall will be discarded. After inspection, the vessel is turned back and coaptation of the cusps are inspected by moving the homograft back and forth in the Ringer-acetate to assess the coaptation area. Second, the vessel is filled up with Ringer-acetate and held up with forceps, to look for valve leakage. Minimal leakage is accepted, if the cusps show proper coaptation. Signs of moderate to severe leakage, prolapse of the cusps, valve insufficiency or anatomical abnormality are reasons for discard of the tissue.

### Recipients

Inclusion criterion for recipients were homograft implantation in the RVOT, with a homograft that was collected at the Tissue Bank in Lund between 1995 and 2018 and implanted during the same time period. Surgery was performed at the pediatric cardiac surgery unit or at the department of cardiothoracic surgery at Skane University Hospital in Lund. In total, 535 homografts were used for 468 different patients. Follow-up of homograft recipients started the day of implantation and finished at December 31st, 2019. Endpoint was defined as homograft intervention due to homograft dysfunction, such as replacement of the homograft, endovascular intervention or other invasive interventions. Some reinterventions were considered non-homograft related, and these patients were censored at reintervention. These interventions include heart transplant, Glenn procedure, total cavopulmonary connection (TCPC) or replacement of the autograft after Ross procedure. In case of heart transplants, patients had complex heart defects, where heart failure was the main reason for reintervention rather than homograft failure. At Glenn and TCPC interventions, patients were undergoing surgery at a specific age no matter the homograft function. In one case, the autograft failed in the aortic position after the Ross procedure. It was replaced by a valve prothesis and the homograft was replaced with the autograft i.e. the native pulmonary valve.

Homograft related death was also considered as endpoint. Patients who died from other causes during follow-up was censored at their time of death.

Nineteen patients were excluded from the study due to moving abroad shortly after surgery. Six patients were excluded since they received their homograft in 2019. Eight patients were lost to follow-up and censored at their last registered visit at the hospital.

Follow-up is conducted at the recipient’s domicile hospital. Decisions on reintervention are made on an individual basis in a multidisciplinary conference. Indications for discussion are decreased physical capacity, progressive right ventricular (RV) dilatation, RV end-diastolic volume > 150 ml/m^2^ body surface area, RV ejection fraction < 45%, pulmonary regurgitation fraction > 40%, pulmonary stenosis with maximal gradient of 50 mmHg or > 4 m/s on Doppler recording, tricuspid regurgitation or ventricular arrhythmias.

### Statistical analysis

Continuous variables are presented as median with interquartile range. Categorical variables are presented with absolute numbers and percentages. *p* value < 0.05 was considered significant. Confidence level was set at 95%.

Freedom from reintervention was analyzed with univariable and multivariable Cox proportional hazard regression. Clinically relevant variables were chosen for the univariable analysis, including presence of structural changes, homograft type, homograft size, donor age, donor type, anatomic position at implantation, recipient age and time era of surgery. Structural changes are defined as “none”, “fenestrations of the cusps” or “fibrosis of the cusps and/or soft atheromatosis of the vessel”. Homograft with both fenestrations and fibrosis and/or atheromatosis of the vessel were included in the fenestration group. Time era of surgery is divided into three groups, with equally sized time eras in each group. The group with the lowest reintervention rate was used as reference group in all variables.

All variables were considered for inclusion in the multivariable model. To avoid multicollinearity, correlation between continuous variables were checked with Pearson’s test of correlation.

A backward stepwise multivariable Cox Proportional Hazard Regression model was conducted. First, all variables except the variable of interest (structural changes of the homograft) was included. In every step, the variable with the highest *p* value was excluded until all variables had a *p* value < 0.1. The variable of interest, “structural changes of the homograft”, was included in the final model.

Analyses was performed with Stata (StataCorp. 2017. Stata Statistical Software: Release 15. College Station, TX, USA: StataCorp LLC).

## Results

### Recipients

Patient characteristics are shown in Table 1. Median age at surgery was 12.6 years (4.9–24.5, range 0–72 years). Median follow-up was 9.9 years (4.8–14.6, range 0.2–24.3 years). Follow-up was 98.4% complete.

**Table 1:** Recipients of homografts in the RVOT, Lund, between 1995 and 2018

	n = 510	%
Recipient age, years		
0–1	55	10.8
1–7	101	19.8
7–18	206	40.4
≥ 18	148	29.0
Diagnosis		
PA^a^, VSD^b^	18	3.5
PA, VSD, MAPCA^c^	23	4.5
TGA^d^, VSD, PS^e^	9	1.8
TA^f^	34	6.7
PA, IVS^g^	12	2.4
Conduit exchange	154	30.2
Fallot^h^	119	23.3
Ross	81	15.9
Other^i^	60	11.8
Gender		
Male	300	58.8
Female	210	41.2
Anatomic position		
Anatomic	287	56.3
Extra-anatomic	223	43.7
Conduit number		
First	356	69.8
Second	118	23.1
Third	30	5.9
Forth	5	1.0
Sixth	1	0.2
Time era of surgery		
1995–2002	143	28.0
2003–2010	237	46.5
2011–2018	130	25.5

^a^Pulmonary atresia

^b^Ventricular septal defect

^c^Major aortopulmonary collateral artery

^d^Transposition of the great arteries

^e^Pulmonary stenosis

^f^Truncus arteriosus

^g^Intact ventricular septum

^h^“Fallot” include patients previously corrected for Teratology of Fallot, who developed postoperative pulmonary insufficiency or stenosis

^i^“Other” include absent pulmonary valve syndrome, isolated pulmonary insufficiency or stenosis, congenital corrected transposition and double-outlet right ventricle with associated pulmonary insufficiency or stenosis

### Mortality

Twenty-four (4.7%) patients died during follow-up. Eleven patients died from cardiac events (heart failure, myocardial infarction or cardiac arrest). Five patients died from unknown causes. Four patients died from malignancy. One patient died from respiratory failure. One patient died from abdominal sepsis. One patient died from respiratory failure 2 month after surgery, due to post-operative complications consisting of cardiac tamponade with cardiac arrest and severe brain damage. One patient died suddenly at home. The autopsy showed rupture of a pseudoaneurysm related to the suture line. The cause of the pseudoaneurysm is unknown, but might be due to surgical technique. Another explanation could be weakness of the muscle cuff of the homograft. The case was closely reviewed, but there were no abnormalities in the collection or processing of the homograft. The time of death was considered to be the end-point of this homograft. All other patients were censored at their time of death.

### Donors and homografts

A total of 2860 homografts were collected and prepared between 1995 and 2018, and 1273 (44.5%) were discarded. Donor and homograft characteristics are presented in Table 2. Missing data are due to incomplete registration of donors and homografts. Main reasons for discard was presence of fenestrations, n = 542 (42.6%) or atheromatosis, n = 322 (25.3%). Description of structural changes is presented in Table 3. Data are not recorded on fenestrations in 301 (10.5%) homografts and atheromatosis in 624 (21.8%) homografts. All these homografts but one is discarded, the last one is from 1995 where some documentation has been lost. When a homograft is discarded due to fenestrations, description of other structural defects is often missing. Same pattern is presented when a homograft is discarded due to atheromatosis, when registration of fenestrations is often missing. There is also an issue with registration on fenestration type and size, which in most cases are described as “minimal”, “small” or “large”, but no exact measurements or locations are registered.

**Table 2:** Donor and homograft characteristics

	All		Discarded		Implanted for RVOT, Lund	
	n = 2860	%	n = 1273	%	n = 510	%
Donor age, years						
0–1	129	4.5	36	2.8	29	5.7
1–15	202	7.1	38	3.0	88	17.3
15–30	564	19.7	205	16.1	141	27.6
30–50	888	31.0	404	31.7	135	26.5
≥ 50	1075	37.6	588	46.2	117	22.9
Missing	2	0.1	2	0.2		
Donor gender						
Male	1680	58.7	796	62.5	293	57.5
Female	1152	40.3	462	36.3	213	41.8
Missing	28	1.0	15	1.2	4	0.8
Donor type						
Multi organ	1119	39.1	492	38.6	151	29.6
Domino	461	16.1	249	19.6	63	12.4
NHBD^a^, 1–24 h	495	17.3	193	15.2	112	22.0
NHBD^a^, > 24 h	767	26.8	321	25.2	184	36.1
Missing	18	0.6	18	1.4		
Homograft type						
Pulmonary	1440	50.3	580	45.6	418	82.0
Aortic	1420	49.7	693	54.4	92	18.0
Homograft size, mm						
0–10	101	3.5	35	2.7	20	3.9
10–20	519	18.1	104	8.2	169	33.1
20–30	1522	53.2	418	32.8	321	62.9
≥ 30	12	0.4	10	0.8		
Missing	706	24.7	706	55.5		

“All” refers to all collected homografts

^a^Non-heart beating donor, divided according to different ischemic times

**Table 3:** Structural changes of the homografts

	All		Discarded		Implanted for RVOT, Lund	
	n = 2860	%	n = 1273	%	n = 510	%
Fenestrations						
No	1620	56.6	282	22.2	472	92.5
Yes	727	25.4	592	46.5	31	6.1
Fibrosis of cusps	203	7.1	90	7.1	7	1.4
Prolapse	7	0.2	7	0.5		
Other^a^	2	0.1	2	0.2		
Missing	301	10.5	300	23.6		

	n = 727	%	n = 592	%	n = 31	%
Fenestration type						
Central	39	5.4	34	5.7	0	0
Peripheral	120	16.5	24	4.1	22	71.0
Missing	568	78.1	534	90.2	9	29.0

	n = 2860	%	n = 1273	%	n = 510	%
Atherosclerosis						
No	1754	61.3	386	30.3	503	98.4
Yes	481	16.8	262	20.6	7	1.4
Other^b^	1	0.1	1	0.1		
Missing	624	21.8	624	49		

“All” refers to all collected homografts

^a^“Other” include one homograft with a vegetation on the cusps and one homograft with cusps that were stuck together. Both were discarded

^b^“Other” include one homograft with an aneurysm, that was discarded

### Follow-up

There were 136 (26.7%) reinterventions and one (0.2%) homograft related death (see “Mortality” section) during follow-up. Of all reinterventions, 110 (80.9%) patients underwent valve replacement with open heart surgery, using a new homograft (n = 79), a Contegra valve (n = 23) or another biological valve (n = 8). Twenty-five (18.2%) patients underwent endovascular intervention with valve replacement (n = 18), balloon dilation (n = 4) or stent insertion (n = 3). One patient (0.7%) underwent resection of adhesions affecting the homograft.

Eight (1.5%) patients were censored at their reintervention. Five patients underwent heart transplant, one patient underwent Glenn procedure, one patient underwent TCPC and one patient got his native pulmonary valve replaced in the RVOT after early dysfunction in the aortic position after a Ross procedure.

Freedom from reintervention was 98.0% (95% CI 96.3–98.9%) at 1 year, 89.2% (95% CI 86.0–91.7%) at 5 years, 75.8% (95% CI 71.3–79.7%) at 10 years, 66.8% (95% CI 61.5–71.6%) at 15 years and 57.4% (95% CI 50.0–64.0%) at 20 years.

Result from the univariable analysis are shown in Table 4. Pearson’s test of correlation showed high correlation between homograft size and donor age (0.71). To avoid collinearity, donor age was excluded from the multivariable model.

**Table 4:** Uni- and multivariable analysis of potential risk factors in relation to time to reintervention after homograft implantation in the RVOT

	Univariable analysis			Multivariable analysis		
	HR^a^	95% CI^b^	*p* value	HR	95% CI	*p* value
Structural changes						
No changes	1.00			1.00		
Fenestration	0.27	0.07–1.10	0.068	0.46	0.11–1.87	0.276
Fibrosus or atheromatosis	0.88	0.28–2.76	0.826	0.80	0.25–2.56	0.704
Homograft type						
Pulmonary	1.00			1.00		
Aortic	4.17	2.95–5.87	< 0.001	1.47	0.99–2.17	0.053
Homograft size	0.80	0.77–0.83	< 0.001	0.88	0.83–0.94	< 0.001
Donor age	0.94	0.93–0.95	< 0.001			
Donor type						
Multi organ	1.00					
Domino	1.92	1.02–3.61	0.042			
NHBD^c^ 1–24 h	2.86	1.68–4.85	< 0.001			
NHBD^c^ > 24 h	1.99	1.20–3.28	0.007			
Anatomic position						
Anatomic	1.00					
Non-anatomic	4.14	2.82–6.08	< 0.001			
Recipient age	0.90	0.88–0.92	< 0.001	0.95	0.93–0.98	0.001
Time era of surgery						
1995–2002	1.12	0.60–2.10	0.712			
2003–2010	0.87	0.47–1.60	0.659			
2011–2018	1.00					

^a^Hazard ratio

^b^Confidence interval

^c^Non-heart beating donor

The result from the final step in the multivariable Cox Proportional Hazard Regression is shown in Table 4. In the last model, homograft structure was included. Only homograft size and recipient age proved to be significant risk factors for reintervention in the multivariable model. Homograft type is borderline significant. Structural changes in the homograft showed no significant impact on the long-term result after homograft implantation (*p* = 0.276 for fenestrations and *p* = 0.704 for fibrosis and/or atheromatosis).

## Discussion

The focus of this study was to evaluate the effect of small fenestrations and other structural changes in the homografts on long-term outcome in the recipient. Our result cannot show any significant impact of structural changes in the homograft on long-term outcome in the recipient. Once again, we show that small homograft size, young recipient age and aortic homografts are risk factors for earlier reintervention of the homograft.

The total discard rate of all collected and prepared homografts in the study period was 45%. Fenestrations, atheromatosis and fibrosis are the main reasons for rejection of homografts at the Tissue Bank in Lund, where 68% of rejected homografts are due to these structural changes. Fenestrations only are the reason in 43%. Other Tissue Bank describe that 42-58% of their discards were due to morphological deterioration (Jashari et al. 2010; Ling Heng et al. 2013; Paolin et al. 2017). Our proportion of discards due to morphological deterioration are higher than these reported numbers, which could be due to different inspection methods. Another aspect might be that the Tissue Bank in Lund has a different practice on discard due to microbiology compared to the EDQM recommendations (Keitel 2019), with a small proportion of rejects due to microbiology (3%). According to Zahra et al. (2019), many tissue banks have microbiological contamination as their main reason for discard. It might be that the absolute numbers of rejects due to structural changes are similar, but the proportion gets higher at the Tissue Bank in Lund due to no other major reasons for discard.

Up to this date, we have not found any other studies that evaluates the effect of structural changes on homograft outcome and performance. Our data show that a very small proportion of homograft with structural changes, including fenestrations, are accepted for homograft implantation. In total, only 9% of the homograft used for RVOT reconstructions in Lund had any kind of structural changes.

Today, we do not accept fenestrations larger than 3 × 2 mm adjacent to the commissures. According to the results in this study, this seem to be acceptable guidelines when using homografts for transplantation. However, our result also shows that current guidelines in our Tissue Bank exclude almost all homografts with structural changes from transplantation. The Tissue Bank in Lund, together with many other tissue banks, cannot always meet the demands from the cardiothoracic departments. Especially pulmonary homografts are often in shortage. It would be relevant to consider if it’s possible to be more liberate when it comes to accepting small fenestrations, but additional studies must be made first. A clear limitation in this study is the size of the group, where only 43 recipients received a homograft with structural changes, and only 6 events occurred in this group. This makes it difficult to draw any major conclusions from the results, even if it seems like small morphological changes does not affect the long-term outcome. It would be of great interest to analyze similar data from other tissue banks in the future. The availability of homografts is always limited, and we should aim for guidelines that allow as many homografts as possible to be used for transplantation, without affecting the quality of tissue.

When looking at reasons for discard in different time eras, reject due to structural changes are less common before 2005, when only a few homografts have structural changes registered as reason for reject (data not shown). In recent years, the importance of strict data registration in tissue banks have been noticed, and major effort has been made to improve the accuracy of the registration in Lund. Discards for structural changes were probably not absent in the early years, but rather these homografts were rejected and not registered at all. The total proportion of discards due to structural changes might be higher than the data shown due to this error. 17.7% of the homografts registered were harvested before 2005.

Except for data registration, other routines have varied through the years between 1995 and 2018. For example, the antibiotic cocktail for decontamination have been altered two times and routines of microscopic examination of tissue have differed through-out the years. Seeing that different time eras did not show any statistical significance on reintervention rate, these routine changes does not seem to impact the long-term outcome in the recipient.

A strength of this study is a large recipient group with up to 24 years of follow-up, where the Tissue Bank in Lund has a close connection with the clinical departments, and good opportunities to follow patients who got homografts implanted in Lund. There is a 1.6% loss to follow-up, but the majority of these patients (89%) where censored in 2015 and later, with only a few years missing from complete follow-up.

## Conclusion

Small fenestrations of the homograft cusps and minor atheromatosis of the homograft vessel wall does not seem to impact the long-term outcome after homograft implantation in the RVOT. Current guidelines on structural deterioration seem to be acceptable, considering homograft quality and durability.

## Data Availability

Raw data and statistical code for Stata, version 15, are available from the corresponding author upon request.

## Abbreviations

EDQM: European Directorate for the Quality of Medicines and Health Care
NHBD: Non-heart beating donors
RV: Right ventricle
RVOT: Right ventricular outflow tract
TCPC: Total cavopulmonary connection

## References

[CR1] Axelsson I, Malm T (2018). Long-term outcome of homograft implants related to donor and tissue characteristics. Ann Thorac Surg.

[CR2] Bielefeld MR, Bishop DA, Campbell DN, Mitchell MB, Grover FL, Clarke DR (2001). Reoperative homograft right ventricular outflow tract reconstruction. Ann Thorac Surg.

[CR3] Boethig D, Goerler H, Westhoff-Bleck M, Ono M, Daiber A, Haverich A, Breymann T (2007). Evaluation of 188 consecutive homografts implanted in pulmonary position after 20 years. Eur J Cardio Thorac Surg.

[CR4] Brown JW, Ruzmetov M, Rodefeld MD, Vijay P, Turrentine MW (2005). Right ventricular outflow tract reconstruction with an allograft conduit in non-ross patients: risk factors for allograft dysfunction and failure. Ann Thorac Surg.

[CR5] Da Costa FDA, Colatusso DF, Filho EMB, Marchetti R, De Aragon Ferreira AD, Da Costa MBA, Roderjan JG, Colatusso C (2017). 20 years experience with the Ross operation in middle-aged patients: the autologous principle is still alive. Interact CardioVasc Thorac Surg.

[CR6] Dekens E, Van Damme E, Jashari R, Van Hoeck B, François K, Bové T (2019). Durability of pulmonary homografts for reconstruction of the right ventricular outflow tract: how relevant are donor-related factors?. Interact CardioVasc Thorac Surg.

[CR7] Gerestein CG, Takkenberg JJM, Oei FBS, Cromme-Dijkhuis AH, Spitaels SEC, Van Herwerden LA, Steyerberg EW, Bogers AJJC (2001). Right ventricular outflow tract reconstruction with an allograft conduit. Ann Thorac Surg.

[CR8] Gröning M, Tahri NB, Søndergaard L, Helvind M, Ersbøll MK, Ørbæk Andersen H (2019). Infective endocarditis in right ventricular outflow tract conduits: a register-based comparison of homografts, contegra grafts and melody transcatheter valves. Eur J Cardio Thorac Surg.

[CR9] Jashari R, Goffin Y, Vanderkelen A, Van Hoeck B, du Verger A, Fan Y, Holovska V, Brahy O (2010). European homograft bank: 20 years of cardiovascular tissue banking and collaboration with transplant coordination in Europe. Transplant Proc.

[CR10] Kalfa D, MacÉ L, Metras D, Kreitmann B (2011). How to choose the best available homograft to reconstruct the right ventricular outflow tract. J Thorac Cardiovasc Surg.

[CR11] Keitel S (2019) Guide to the quality and safety of tissues and cells for human application. In: European Committee on Organ Transplantation, 4th edn. Strasbourg, pp 249–254

[CR12] Ling Heng W, Albrecht H, Chiappini P, Phang Lim Y, Manning L (2013). International heart valve bank survey: a review of processing practices and activity outcomes. J Transplant.

[CR13] Meyns B, Jashari R, Gewillig M, Mertens L, Komárek A, Lesaffre E, Budts W, Daenen W (2005). Factors influencing the survival of cryopreserved homografts. The second homograft performs as well as the first. Eur J Cardio Thorac Surg.

[CR14] Paolin A, Trojan D, Petit P, Coato P, Rigoli R (2017). Evaluation of allograft contamination and decontamination at the Treviso Tissue Bank Foundation: a retrospective study of 11,129 tissues. PLoS ONE.

[CR15] Romeo JLR, Mokhles MM, Van De Woestijne P, De Jong P, Van Den Bosch A, Van Beynum IM, Takkenberg JJM, Bogers AJJC (2019). Long-term clinical outcome and echocardiographic function of homografts in the right ventricular outflow tract. Eur J Cardio Thorac Surg.

[CR16] Skoglund K, Svensson G, Thilén U, Dellborg M, Eriksson P (2017). Long-term outcome after right ventricle to pulmonary artery conduit surgery and reintervention. Scand Cardiovasc J.

[CR17] Van Dijck I, Budts W, Cools B, Eyskens B, Boshoff DE, Heying R, Frerich S, Vanagt WY (2015). Infective endocarditis of a transcatheter pulmonary valve in comparison with surgical implants. Heart.

[CR18] Vitanova K, Cleuziou J, Horer J, Kasnar-Samprec J, Vogt M, Schreiber C, Lange R (2014). Which type of conduit to choose for right ventricular outflow tract reconstruction in patients below 1 year of age?. Eur J Cardio Thorac Surg.

[CR19] Zahra S, Galea G, Jashari R, Petit P, de By TMMH (2019). Significant variation in heart valve banking practice. Eur J Clin Microbiol Infect Dis.

